# Interspecific Differences in Metabolic Rate and Metabolic Temperature Sensitivity Create Distinct Thermal Ecological Niches in Lizards (*Plestiodon*)

**DOI:** 10.1371/journal.pone.0164713

**Published:** 2016-10-19

**Authors:** Charles M. Watson, Warren W. Burggren

**Affiliations:** 1 Department of Biology, Midwestern State University, Wichita Falls, Texas, United States of America; 2 Department of Biological Sciences. The University of North Texas, Denton, Texas, United States of America; University of Tasmania, AUSTRALIA

## Abstract

Three congeneric lizards from the southeastern United States (*Plestiodon fasciatus*, *P*. *inexpectatus*, and *P*. *laticeps*) exhibit a unique nested distribution. All three skink species inhabit the US Southeast, but two extend northward to central Ohio (*P*. *fasciatus* and *P*. *laticeps*) and *P*. *fasciatus* extends well into Canada. Distinct interspecific differences in microhabitat selection and behavior are associated with the cooler temperatures of the more Northern ranges. We hypothesized that interspecific differences in metabolic temperature sensitivity locally segregates them across their total range. Resting oxygen consumption was measured at 20°, 25° and 30°C. *Plestiodon fasciatus*, from the coolest habitats, exhibited greatly elevated oxygen consumption compared to the other species at high ecologically-relevant temperatures (0.10, 0.17 and 0.83 ml O_2_^.^ g^-1.^ h^-1^ at 20°, 25° and 30°C, respectively). Yet, *P*. *inexpectatus*, from the warmest habitats, exhibited sharply decreased oxygen consumption compared to the other species at lower ecologically-relevant temperatures (0.09, 0.27 and 0.42 ml O_2_^.^ g^-1.^ h^-1^ at 20°, 25° and 30°C, respectively). *Plestiodon laticeps*, from both open and closed microhabitats and intermediate latitudinal range, exhibited oxygen consumptions significantly lower than the other two species (0.057, 0.104 and 0.172 ml O_2_^.^ g^-1.^ h^-1^ at 20°, 25° and 30°C, respectively). Overall, *Plestiodon* showed metabolic temperature sensitivities (Q_10_s) in the range of 2–3 over the middle of each species’ normal temperature range. However, especially *P*. *fasciatus* and *P*. *inexpectatus* showed highly elevated Q_10_s (9 to 25) at the extreme ends of their temperature range. While morphologically similar, these skinks are metabolically distinct across the genus’ habitat, likely having contributed to their current distribution.

## Introduction

Ecological physiologists have long questioned how species distribution is affected by local environment, phenotype and phylogenesis. Yet, few unifying principles have emerged. The five-lined skink lizards of the forests in the eastern United States comprise a highly tractable system to investigate these phenomena, given that they share close phylogenetic relationships, phenotypic similarities, regional sympatry, and latitudinally-stratified northern range boundaries. Among the skinks, the common five-lined skink (*Plestiodon fasciatus*), the broadhead skink (*Plestiodon laticeps*) and the southeastern five-lined skink (*Plestiodon inexpectatus*) are phenotypically similar during much of their development and also exhibit similarities in behavior and diet [1, 2]–so much so they were considered a single species prior to their formal designation as distinct species [3–5]. Adult *P*. *laticeps* represent an obvious exception to these generalities because they are considerably larger than adult *P*. *fasciatus* and *P*. *inexpectatus*, thus allowing them to exploit different resources [2, 6, 7]. The ecological physiology of these closely related species is still poorly known, but they are of interest because they occur in regional sympatry and may be using the same set of resources. This is an apparent violation of both the competitive exclusion hypothesis and Jordan’s Rule, which states that an organism’s closest relative would be found either in adjacent, dissimilar habitats or separate but similar habitats [8–10]. While an important point to consider, this contravention of Jordan’s Rule may be irrelevant if one considers fine scale differences in microhabitat rather than broader habitat generalizations.

Dimensions of the ecological niches (e.g. thermal, spatial, and temporal) of these *Plestiodon* species have not been shown to differ experimentally. However, environmental temperature has been implicated as a potential factor differentiating the niches of these species [9, 11], for three reasons. First, these species exhibit a latitudinal stratification in northernmost range limits. *P*. *inexpectatus* is restricted to the warmer southeastern United States, while *P*. *laticeps* extends northward to around 40° latitude, and *P*. *fasciatus* extends northward into Canada [2]. Secondly, *P*. *fasciatus* is a deep forest dweller in the warmer south, but occupies open meadows and forest edges in the cooler northern parts of its range [7] [12]). Lastly, in areas of syntopy, field observations indicate that *P*. *inexpectatus* inhabits more open, warmer, microhabitats than the other two species [9, 11]. Preferred temperatures of these species range from ~32.5°C (*P*. *fasciatus*) to ~34°C (*P*. *laticeps*) [11]. Further details of the range and habitat of these lizards can be found in Conant and Collins [2].

Despite these marked differences in the thermal characteristics of the habitats of various species of *Plestiodon*, no measurements of metabolic rate and its sensitivity to temperature have been made across a North American cohort of species for this genus. Consequently, we hypothesize that differences in preferred microhabitat temperature among these closely related lizard species are correlated with intrinsic differences in thermal sensitivity of their metabolic rate. We additionally hypothesize that such differences could explain the physiological constraints underlying the temperature-related limitations of these species’ range, as previously described [9]. To test these hypotheses, we measured how these species responded metabolically to a range of temperatures between 20°C and 30°C, which encompasses their range of measured field temperatures. Our findings indicate that there are distinct differences in metabolic temperature sensitivity over the tested ranges, and that these differences help explain the patterns of species distribution.

## Materials and Methods

### Animal Collection

Lizards were collected during the spring and summer of 2006 and 2007 throughout the Southeastern United States from areas where all three species are regionally sympatric. Specimens were collected from a relatively constrained latitudinal range (~30°N-33.5°N) in order to negate the potentially confounding effects of latitudinal acclimatization as described for *Sceloporus* [13].

Animal collection and experimentation was conducted under protocols #A06.017 and #A07.003 approved by the IACUC of the University of Texas at Arlington.

### Animal Maintenance

Individuals were maintained in the laboratory for a minimum of 1 month prior to the beginning of metabolic (oxygen consumption) measurements. During this maintenance period, animals were kept in commercially-available plastic boxes with a shredded aspen substrate, a water bowl, and a ceramic tile for refuge. They were maintained with a 12-hour light/dark cycle (on at 0800h and off at 2000h CST) at room temperature, which uniformly fluctuated between 22.5–27.0°C. No thermal gradients were evident in the holding cages. Three to five crickets were offered twice weekly. All work was performed under The University of Texas at Arlington IACUC protocols #A06.017 and #A07.003.

### Temperature Acclimation and Oxygen Consumption Measurement Protocol

**Fig 1:**
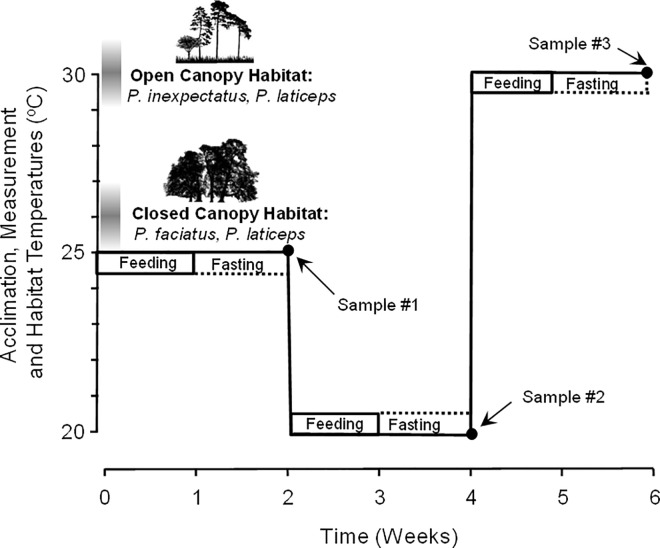
Protocol for temperature acclimation for oxygen consumption measurements in *Plestiodon fasciatus*, *Plestiodon inexpectatus*, and *Plestiodon laticeps*. The left side of the panel depicts the daily habitat temperature range of the three species (after [9, 11], while the right side shows the time course of the temperature acclimation and sampling schedule for the measurements of resting oxygen consumption.

After the preliminary maintenance period in the laboratory, lizards in their enclosures were placed inside a walk-in environmental chamber with a 12 hour light/12 hour dark light cycle. There are a variety of protocols for subjecting animals to variation in ambient temperature, each with advantages and disadvantages. For example, temperature can be first raised, then lowered (or vice versa), or temperature can be changed in a stepwise or ramp fashion. Metabolic rate can be affected by the previous high or low acclimation temperature. There is no “standard” protocol for such experipmetns. During the course of animal husbandry, we determined that *P*. *fasciatus* may be especially sensitive to high temperature. Consequently, in this study, lizards were subjected to a rigidly controlled temperature acclimation and oxygen consumption measurement protocol where lizards were exposed to the highest temperature last (Fig 1). All lizards of each of the three species were first acclimated to 25±<0.5°C for two weeks (Fig 1), at the end of which their oxygen consumption ($\dot{V}O_{2}$) was individually measured (see below) and they were weighed. The temperature was then lowered to 20°C for two weeks, followed by a second $\dot{V}O_{2}$ measurement and weighing. The temperature was then raised to 30°C for a final period of two weeks, finishing with a final $\dot{V}O_{2}$ measurement and weighing. All measurements were made between 1500h and 1900h CST. The temperature range of 20–30°C for laboratory measurements was specifically selected for three reasons: 1) 20°C reflects the low ambient temperature of the general habitat of all species during the seasons in which they are active, 2) 25°C approximates the mean ambient daily temperature of the habitat frequented by *Plestiodon fasciatus* and *P*. *laticeps*, and 3) 30°C approximates the mean ambient daily summer temperature of the microhabitat frequented by *P*. *inexpectatus* and *P*. *laticeps* (30°C) [9, 11]. The acclimation and measurement temperatures, as well as the field temperatures determined from the studies indicated above, are shown on Fig 1 for the two habitats.

Specific dynamic action–the increase in metabolic rate after eating–can be both large and long-lasting in reptiles [14–17]. To avoid complex interactions of thermal acclimation and specific dynamic action, all lizards were fasted for one week prior to each experimental $\dot{V}O_{2}$ trial (i.e., for the second half of the acclimation period, as indicated in Fig 1) Lizards were provided water *ad libitum* throughout the acclimation/measurement protocol.

### Respirometry

Resting $\dot{V}O_{2}$ values were measured via standard closed-system respirometry techniques described for terrestrial and aquatic animals [18–20]. Each lizard was placed within a 500 ml Erlenmeyer flask that was sealed with a gas-tight rubber stopper. Verification of air-tightness of the respirometry system was initially verified by submerging the sealed respirometer in water and inspecting for escaping air bubbles. Two ports in the stopper, an inlet and outlet, were each fitted with glass tubing and a three way stopcock. CO_2_ absorbant (Ascarite) in a small porous container was placed in each respirometer. The flasks were covered with aluminum foil to visually shield the lizards from the researchers. Movement of the lizard when first placed within the respirometer could be audibly detected by the researchers, but animals settled down within minutes with no further evidence of movement. Thus, it was assumed that the lizards were at rest during these trials. The respirometers, oxygen analysis equipment and acclimating animals were placed inside the environmental chamber. Each animal was placed from their temperature acclimation enclosure directly into a respirometer for 1 h, with air being pumped through the respirometer using a commercially available aquarium air pump. After this period of acclimation to the respirometer, the barometric pressure (in mmHg) was measured and the respirometers were sealed for $\dot{V}O_{2}$ measurement.

Lizards were left in the closed respirometers for 1–3 h, depending upon body size. Larger lizards use more oxygen at the same metabolic rate as smaller specimens due to the larger volume of respiring tissue. This effect is exacerbated by the lowered amount of gas available in the respirometer because of larger body size, resulting in a reduced volume of airavailable to breath. Therefore, larger specimens took less time to depleat the air of oxygen and produce a measurable result. Preliminary experiments revealed that this was a sufficient time period to reduce PO_2_ in the chambers by ~8–12 mmHg. This change was large enough to be measured accurately, but not so large as to cause significant hypoxia in the chambers. Upon the completion of each respirometry trial, which lasted from 1–3 h, a 4 ml sample of gas from inside the respirometer was drawn via a stopcock into a gas-tight glass syringe and then injected into a thermostatted PO_2_ electrode connected to a Radiometer PHM72 gas meter. Each animal was then weighed. $\dot{V}O_{2}$ was calculated using standard closed respirometry equations [18, 20, 21], with the following variables: decline in PO_2_ in the sealed respirometer over a known elapsed period of time, volume of the chamber, volume of the animal, and temperature and barometric pressure of gas in the respirometer. Mass-specific resting $\dot{V}O_{2}$ was expressed as ml O_2_^.^ g^-1.^ h^-1^ (STP).

Temperature coefficient (Q_10_) values for $\dot{V}O_{2}$ were calculated for the temperature intervals of 20–25°C and 25–30°C using the Van’t Hoff equation:

$$Q_{10}={\left(\frac{R_{2}}{R_{1}}\right)}^{\left(\frac{10}{T_{2}-T_{1}}\right)}$$

where *R*_*1*_ *and R*_*2*_ is the $\dot{V}O_{2}$ at *T*_*1*_ *and T*_*2*_, respectively.

### Data Analysis and Statistics

Resting $\dot{V}O_{2}$ values were log-transformed to satisfy the assumption of normality. A One-way repeated measures ANOVA was carried out using species and acclimation temperature as factors. A post hoc Tukey test was used to determine specific differences among species at each acclimation temperature (α = 0.05). Q_10_ values were log transformed to satisfy the assumption of normality and analyzed with a one-way repeated measures ANOVA performed to test the differences among species at each of the two intervals. A post-hoc Fisher LSD test was used to determine significance among species (α = 0.05). All data are expressed as $\bar{x}$ ± SE unless otherwise stated.

## Results

### Species-Specific Body Mass and Size

**Fig 2:**
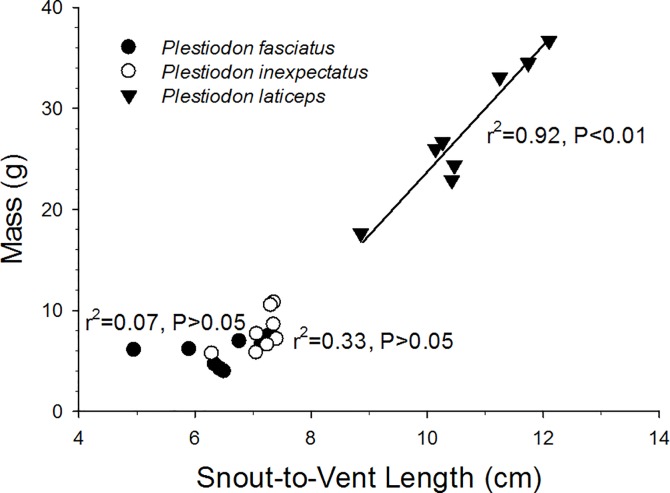
Size distribution, with individual regression lines for adult *Plestiodon fasciatus*, *P*. *inexpectatus*, and *P*. *laticeps* used to determine temperature sensitivity of oxygen consumption (Q_10_). r^2^ and P values are indicated for each species–only *P*. *laticeps* showed a significant relationship between body mass and snout-to-vent length.

The distribution of mass and snout-to-vent length (SVL) of the three species of *Plestiodon* used in this study is presented in Fig 2. Adult *Plestiodon laticeps* were significantly longer and heavier (10.55 ± 0.36 mm, 27.73 ± 2.30 g) than adult *P*. *fasciatus* (6.41 ± 0.36 mm, 5.80 ± 0.47 g) and *P*. *inexpectatus* (10.55 ± 0.36 mm, 7.87 ± 0.69 g). There is extensive overlap in both mass and snout-to-vent length in the two smaller species. Only in *P*. *laticeps* was the relationship between mass and SVL significant. Thus, there are relatively large ranges in body length for adults of similar body mass in *P*. *fasciatus* and *P*. *inexpectatus* (Fig 2).

### Resting Oxygen Consumption

**Fig 3:**
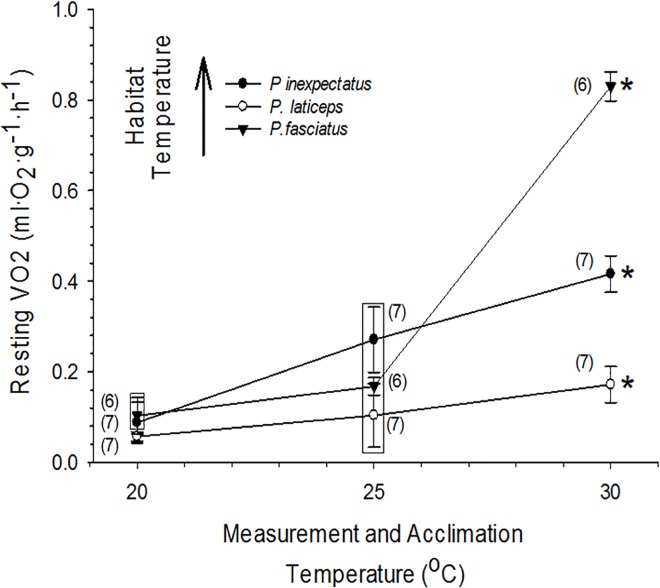
Resting oxygen consumption at 20°, 25° and 30°C for the scincid lizards *Plestiodon fasciatus*, *Plestiodon inexpectatus*, and *Plestiodon laticeps*. Means ± 1 SE and n (in parentheses) are given. An * indicates significant (P<0.05) temperature affect for each species. Boxes enclose statistically identical mean values at each temperature.

Resting $\dot{V}O_{2}$ at 20°C was approximately 0.06–0.1 mlO_2_^.^g^-1.^h^-1^ in all three species (Fig 3). The resting $\dot{V}O_{2}$ of *P*. *laticeps* was slightly but significantly lower than that of *P*. *fasciatus* and *P*. *inexpectatus* at 20°C. In fact, this trend continued at all acclimation/measurement temperatures, where the $\dot{V}O_{2}$ of *P*. *laticeps* was consistently significantly lower than that of *P*. *fasciatus* and *P*. *inexpectatus*.

Not unexpectedly, $\dot{V}O_{2}$ increased with increasing temperature in all three species. However, as temperature increased, the three species showed distinctly different trends in rising $\dot{V}O_{2}$ (Fig 3). Resting $\dot{V}O_{2}$ of *P*. *laticeps* increased the least of the three species, rising from 0.057 ml O_2_^.^g^-1.^h^-1^ at 20°C to just 0.172 ml O_2_^.^g^-1.^h^-1^ at 30°C, a 2.3-fold increase. Over that same 10°C temperature range, the $\dot{V}O_{2}$ of *P*. *inexpectatus* rose from 0.088 ml O_2_^.^g^-1.^h^-1^ at 20°C to 0.416 ml O_2_^.^g^-1.^h^-1^ at 30°C, a 3.7-fold increase. *P*. *fasciatus* showed the greatest sensitivity of metabolism to temperature, with $\dot{V}O_{2}$ rising from 0.103 ml O_2_^.^g^-1.^h^-1^ at 20°C to 0.830 ml O_2_^.^g^-1.^h^-1^ at 30°C, a very large 7.1-fold increase (Fig 3). This large temperature-stimulated increase in metabolism in *P*. *fasciatus* was primarily the result of a large increase in $\dot{V}O_{2}$ from 25°C to 30°C, since *P*. *fasciatus* and *P*. *inexpectatus* exhibited no significant difference (P>0.10) in $\dot{V}O_{2}$ at either 20°C or 25°C. Indeed, at 30°C, resting $\dot{V}O_{2}$ was significantly different in all three species, with $\dot{V}O_{2}$ showing the following relationship: *P*. *fasciatus* > *inexpectatus* > *P*. *laticeps* (Fig 3).

### Metabolic Temperature Sensitivity

**Fig 4:**
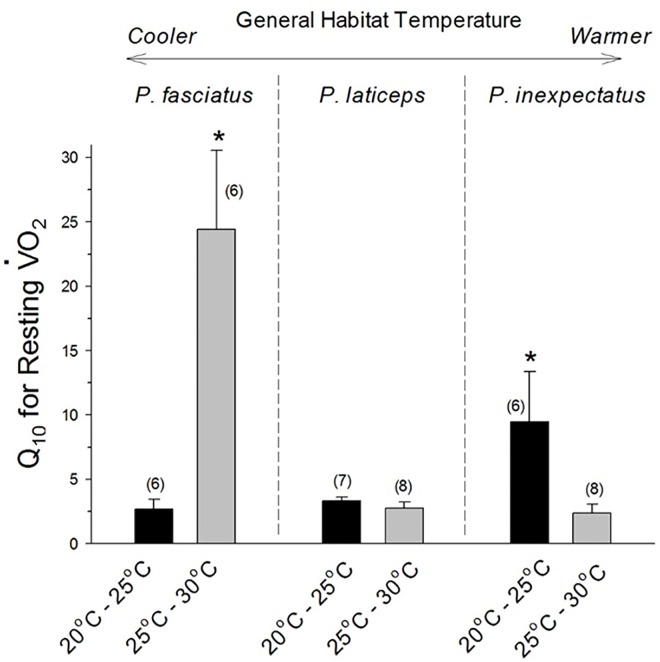
Q_10_ values for resting oxygen consumption for the scincid lizards *Plestiodon fasciatus*, *Plestiodon inexpectatus*, and *Plestiodon laticeps*. The placement of each species is relative to the general habitat temperatures, indicated at the top of the graph. Thus, *P*. *fasciatus* experiences the coolest temperatures, and *P*. *inexpectatus* the warmest. The gray bars show the metabolic response to an increase in temperature from 25°C to 30°C, while the black bars show the metabolic response to a decrease in temperature from 25°C to 20°C. Means ± 1 SE and n (in parentheses) are given. An * indicates significant difference from the other species for Q_10_ for the temperature interval to the right of the graph.

We determined the metabolic temperature sensitivities of each species over the temperature range 20–25°C and 25–30°C, as expressed from the Q_10_ value calculated for $\dot{V}O_{2}$ from the Van’t Hoff equation (Fig 4, see Methods).

Overall, each species exhibited Q_10_ values for $\dot{V}O_{2}$ in the range of 3–4 over the middle of their normal temperature range. *P*. *laticeps*, the species inhabiting the widest temperature range, maintained a relatively constant Q_10_ for both temperature intervals of 20–25°C and 25–30°C, with values of 4.1±0.4 (N = 7); and 3.3 ± 0.6 (N = 8) respectively. The other two species exhibited similar Q_10_ values toward the middle of their typical temperature range. Thus, *P*. *fasciatus*, which is found in cooler habitats, had a Q_10_ at 20–25°C of 3.9±1.2 (N = 6), while *P*. *inexpectatus*, which inhabits warmer habitats, had a Q_10_ of 3.2±1.0 (N = 7) at 25–30°C (Fig 4).

To understand how metabolic responses to temperature might be a determining factor in energy expenditure and thus species range, we also measured Q_10_ at the extremes of the typical temperature ranges for the three species. Both *P*. *inexpectatus* and *P*. *fasciatus*, the two species with the narrowest temperature ranges, exhibited extremely high and variable Q_10_ values towards these temperature extremes. Thus, at the lower range of acclimation and measurement temperatures of 20–25°C, *P*. *inexpectatus* (the species typically dwelling in warmer habitats), exhibited a Q_10_ of 9.5 ± 3.9. On the other hand, *P*. *fasciatus* (typically dwelling in cooler habitats) at the higher range of acclimation and temperature measurements of 25–30°C had a very high Q_10_ *of* 24.4 ± 6.2. Importantly, *P*. *fasciatus* exposed to 30°C for the 14-day acclimation period often refused food over the subsequent few weeks, lost weight, and eventually died over the course of a month or two. However, *P*. *inexpectatus* and *P*. *laticeps* were not adversely affected by the highest acclimation temperature.

## Discussion

### Size Relationships

*Plestiodon laticeps* is the most phenotypically distinct of the three *Plestiodon* species examined in this study. Individuals of *P*. *laticeps* are much larger (Fig 2) and exhibit a greater degree of sexual dimorphism [2, 5]. Furthermore, *P*. *laticeps* exhibits a statistically much stronger linear relationship between mass and snout-to-vent length compared to the other two species. These values are consistent with published maximum sizes for each species. For example, *P*. *laticeps* (SVL: 14.3cm) is more than 60% larger than both *P*. *fasciatus* (SVL: 8.6cm) and *P*. *inexpectatus* (SVL: 8.9cm) [2] and present study). These data are consistent with the much debated Hutchinsonian ratio, which is the body size ratio (1.3:1) that would theoretically allow two similar species to coexist [22]. The ratio of *P*. *inexpectatus’* maximum SVL to *P*. *fasciatus’* maximum SVL is only 1.03:1, at odds with Hutchinson’s ratio. Owing primarily to this difference in adult size, *P*. *laticeps* may not actually be in direct competition with the other two species and indeed *P*. *laticeps* may be partitioning their niche along different dimensions such as preferred prey, space, or microhabitat. This could potentially explain the observation that *P*. *laticeps* is commonly found in sympatry with each of the two smaller species [9, 11]. However, the large amount of size overlap by *P*. *fasciatus* and *P*. *inexpectatus* suggests that they may be in more direct competition for resources and therefore must partition their respective niche spaces. Therefore, physiological differences associated with temperature change may play an important role in niche segregation between these two species.

### Resting Oxygen Consumption

Considerable variation in resting mass-specific $\dot{V}O_{2}$ exists in scincid lizards (Table 1 and S1 Table). Some of this variation may result from size differences, age differences and seasonal changes in metabolic rate. Additionally, prandial state has a major effect on metabolic rate as a result of specific dynamic action in many lizard species Additionally, and beyond scincid lizards, habitat has a notable effect on metabolic rate. For example, desert geckos in the genus *Rhoptropus* have unusually low mass-specific metabolic rates, which may correlate with both their habitat and life style [23]. Resting $\dot{V}O_{2}$ in *Plestiodon*, ranging between 0.1 and 0.3 ml O_2_^.^ g^-1.^ h^-1^ at 20°C, is consistent with values of resting oxygen consumption reported at comparable temperatures for other similarly sized lizards (Table 1), including mostly forest dwelling lizards such as *Eulamprus tympanum* [13], *Eulamprus quoyii* [24], *Sceloporus undulatus* [25, 26], *Eremia lugubris* [27] and *Eumeces chinensis* (now *Plesiodon chinensis*) [28].

**Table 1:** Mass-specific oxygen consumption in scincid lizards.

Lizard		Body Mass(g)	Measurement Temperature (°C)	Mass-Specific Oxygen Consumption(ml^.^ g^-1.^ h^-1^)	Q_10_ (Across Maximum Reported Temperature Range)	Reference
Genus	Species					
*Plestiodon*	*chinensis*	21–38	30	5.0–9.0	-	[28]
	*fasciatus*	5.0–7.3	20, 25, 30	0.10, 0.17, 0.83	8.3	Present Study
	*inexpectatus*	6.0–7.5	20, 25, 30	0.09, 0.27, 0.42	4.7	Present Study
	*laticeps*	9.0–12.2	20, 25, 30	0.06, 0.10, 0.17	2.8	Present Study
*Eulamprus*	*quoyii*	28.8	30	0.12	-	[24]
	*tympanum*	Not reported	30	0.21	-	[13]
*Eremia*	*lineocellata*	3.3	26, 37	0.10, 0.25	2.0	[27]
	*lugubris*	3.8	26, 34, 37	0.09, 0.21, 0.24	2.4	[27]
*Oligosoma*	*nigriplantare polychroma*	3.3	13	0,05*	-	[29]
	*zelandicum*	3.8	13	0.04*	-	[29]
*Cyclodina*	*aenea*	2.5	13	0.05*	-	[29]
	*macgregori*	17.6	13	0,04*	-	[29]
*Scincella*	*lateralis*	0.9	24, 28, 32, 36	0.11, 0.19, 0.19, 0.27*	2.1–3.3	[30]

*values calculated from reported means for body mass and whole animal oxygen consumption.

Interestingly, despite species-specific differences in body size,range and habitat characteristics (including temperature) in the species used for the present study, all three species of *Plestiodon* showed similar levels of $\dot{V}O_{2}$ when measured at 25°C. Moreover, even at higher or lower temperatures, $\dot{V}O_{2}$ of the three species showed similar rates of metabolism (except for *P*. *fasciatus* at 30°C).

Mass is, of course, an important “driver” of metabolic rate in animals, including lizards–for recent reviews see [31–33]. Scaling effects on metabolism have been studied in lizards. For example, a recent study on the agamid lizard *Uromastyx philbyi* [34] has shown profound effects of body mass (and, of course, temperature) on mass-specfic metabolic rate. However, the lizards used in that study varied in size by more than 1 log cycle (~15–175 g), making allometric (scaling) analysis and interpretation more feasible. In the present study the very small size range between individuals within a species and even between species of *Plestiodon* precludes meaningful allometric analysis. Future studies should involve measurement of metabolic rate in a larger range of individuals (e.g. newly hatched and juvenile individuals as well as adults), with the caveat that this will introduce ontogeny as well as body size into the factors affecting mass-specific metabolic rate.

### Interspecific Differences in Metabolic Temperature Sensitivity

Many diurnal lizards have been reported to show Q_10_ values of ~ 2–2.5 [30, 35, 36]. However, closer examination of the metabolic temperature sensitivity reveals that there several factors affecting Q_10_, including season, nutritional state and proximity of the measurement temperature to the animals preferred body temperature. Metabolic thermal sensitivity in *Plestiodon*, as reflected in the Q_10_ for $\dot{V}O_{2}$, ranged from approximately 3.1 to 3.9 when measured in the middle of the normal temperature range for *P*. *fasciatus*, *P*. *inexpectatus* and *P*. *laticeps* (Fig 4). These values are similar to those reported for the desert skink *Scincus mitranus*, whose Q_10_ values ranged from 2.5–3.8 [37] and the temperate skink *Scincella lateralis*, with reported overall values of ~3.5 (though this is an average of Q_10_s measured at both low and high temperatures–see below [30]. Q_10_s ranging from 1.37–8.58, 0.98–2.56 and 2.6–2.8 were also reported for *Acanthodactylus boskianus* and *Diplometopon zarudnyi* [37] andthe zebra-tailed lizard *Callisaurus draconoides* [38]. Generally, the present values are within the high end of the normal published range of temperature sensitivity for resting metabolic rate exhibited by vertebrate poikilotherms [39–44].

Changes in metabolic Q_10_ within a species’ tolerable temperature range can be both greatly exaggerated and highly non-linear, especially when measurements are made towards the extremes of its normal temperature range [30, 41, 43, 45]. Frequently, the relationship of metabolic Q_10_ to temperature is described by a U-shaped curve, with the lowest temperature sensitivity (lowest Q_10_) occurring near the animal’s preferred temperature. Although the present study did not examine metabolic Q_10_ over the entire range of tolerable temperatures for each species of *Plestiodon*, high Q_10_ values at extreme temperatures (for the species) were evident in both *P*. *fasciatus* (from cooler habitats) exposed to high temperatures and *P*. *inexpectatus* (from warmer habitats) exposed to lower temperatures (Fig 4). These metabolic temperature sensitivities are further supported by our personal observations that *P*. *fasciatus* and *P*. *laticeps* remained alert when transported at ~12–13°C, while *P*. *inexpectatus* became flaccid and visibly slowed its breathing at this cooler transport temperature. Similar data have been reported for the scincid lizard *Scincella lateralis* [37]. Metabolic rate in that species, when measured during both summer and winter, was most temperature sensitive between 24 and 28°C (Q_10_ = 5.8), but nearly temperature insensitive between 28 and 33°C (Q_10_ = 1.1).

In the present study, prolonged exposure of *Plestiodon fasciatus* to 30°C temperatures caused individuals of this species to quit eating and eventually die. This laboratory finding would seem contradictory with the fact that body temperatures of basking *P*. *fasciatus* often exceed 30°C in the field [8] (Watson, C. pers. obs.) and that their preferred temperature–acutely—has been experimentally quantified at 32.6°C [8], well above the highest acclimation temperature used in this study. This is also inconsistent with the findings for two nocturnal gecko species, *Ptylodactylus hasselquistii* and *Bunopus tuberculatus*, which exhibited a low metabolic thermal dependence at temperatures that approximate their preferred body temperatures [46]. However, *P*. *fasciatus* is a diurnal, actively-thermoregulating species in a habitat with limited basking opportunities [47]. Consequently, *P*. *fasciatus* almost certainly experiences different selection pressures in native environments. The closed canopy forest habitat of this species dictates that *P*. *fasciatus* must take advantage of direct solar radiation and associated elevated temperatures as a limited resource, so periodic and rapid elevation of metabolism via a shortened basking time may hold a fitness benefit. Brief, periodic basking would temporarily raise the body temperature to or above 30°C, allowing normal metabolic processes to take place while reducing time spent basking and the cost associated with time spent exposed to potential predators. However, prolonged exposure to this temperature appears physiologically untenable, even as it is acutely beneficial.

### Ecological Implications of Interspecific Differences in Metabolic Thermal Sensitivity

*Plestiodon laticeps* can be found in sympatry with *P*. *fasciatus and P*. *laticeps*, but *P*. *fasciatus* and *P*. *inexpectatus* are rarely found together [9, 11]. This distribution could be explained, at least in part, by the metabolic thermal sensitivity of each species, as reflected in the metabolic Q_10_ patterns presented in the current study. Thus, *P*. *fasciatus* may be excluded from areas inhabited by *P*. *inexpectatus* that remain above the former’s critical temperatures for an extended period of time. *P*. *inexpectatus* and *P*. *laticeps*, which are metabolically stable at those same warm temperatures, could remain active throughout the day without having to continually bask. Conversely, *P*. *inexpectatus* would be excluded from those cooler habitats that exhibit low availability of higher temperatures (*i*.*e*. closed-canopy forest), while *P*. *fasciatus* and *P*. *laticeps* would be able to maintain activity in those microhabitats by remaining active with less time spent thermoregulating.

Established geographic distributions [2,7] suggest that the metabolic temperature sensitivity of these species strongly reflects the temperature characteristics of the local niche that each occupies, and may be an important mechanism underlying their distributions. Indeed, the concept of “metabolic niche” has been used to describe broad distributions of endotherms [13] and these findings support the application of this concept. Here, we extend this concept to include the occupation of metabolic niches of ectotherms based upon their metabolic responses to temperature. These findings support our hypotheses as all three species inhabit microhabitats that reflect their metabolic response to temperature and the two like-sized species show significantly different temperature responses that could limit their ability to co-exist. These conclusions depend upon data from sympatric populations, but it may be interesting in future studies to determine whether patterns of character release and/or displacement may occur.

Generally, metabolic rates are species-specific and, for ectothermic organisms, will vary across temperatures. The preferred temperature required to maintain a metabolic rate conducive to homeostasis therefore also varies among species. In the case of *Plestiodon fasciatus* and *P*. *inexpectatus*, there is a measurable difference in metabolism that corresponds to relatively fine-scale temperature differences between open and closed canopy habitats [9, 11]. Although they exhibit morphological similarities that have historically caused them to be considered a single species, the current study demonstrates *P*. *fasciatus* and *P*. *inexpectatus* to differ in their metabolic physiology. These differences could explain a mechanism by which they may be segregated in time and space across the landscape, therefore maintaining an allopatric relationship at the microhabitat scale while remaining regionally sympatric. Therefore, *P*. *fasciatus* and *P*. *inexpectatus* do not appear to be in conflict with the “competitive exclusion hypothesis” [48]. Furthermore, this highlights an instance where differential metabolic responses to temperature among closely-related organisms can possibly structure species’ distributions in a habitat that is thermally dynamic at the local scale.

## Supporting Information

S1 Table Mass and mass-specific oxygen consumption rate for all specimens and trials. (DOCX)

## References

[1] Hamilton WJ, Pollack JA. The food of some lizards from Fort Benning, Georgia. Herpetologica. 1961;17(2):99–106.

[2] Conant R, Collins JT. A Field Guide to Reptiles and Amphibians of Eastern Central North America. New York: Houghton Mifflin; 1998. 616 p.

[3] Taylor EH. *Eumeces inexpectatus*: A new American lizard of the family Scincidae. The University of Kansas Science Bulletin. 1932;20:251–8.

[4] Taylor EH. *Eumeces laticeps*: a neglected species of skink. The University of Kansas Science Bulletin. 1932;20:263–72.

[5] Taylor EH. A taxonomic study of the cosmopolitan scincoid lizards of the genus *Eumeces* with an account of the distribution and relationships of its species. The University of Kansas Science Bulletin. 1935;23:1–643.

[6] Davis D. A study of the variation in North American lizards of the *fasciatus* group of the genus *Eumeces* (Scincidae): Duke University; 1968.

[7] Mount RH. The Reptiles and Amphibians of Alabama. Auburn, AL: Auburn University Agricultural Experiment Station; 1975. 347 p.

[8] Fitch HS. Life history and ecology of the five-lined skink, *Eumeces fasciatus*. University of Kansas Publications of the Museum of Natural History. 1954;8:1–156.

[9] Watson CM, Gough L. The role of temperature in determining distributions and coexistence of three species of *Plestiodon*. J Therm Biol. 2012;37:374–9.

[10] Jordan DS. The law of geminate species. Am Nat. 1908;42:73–80.

[11] Watson CM, Formanowicz DR. A comparison of maximum sprint speed among the five-lined skinks (*Plestiodon*) of the Southeastern United States at ecologically relevant temperatures. Herp Conserv Biol. 2012;7(1):75–82.

[12] Harding JH, Holman JA. Michigan Turtles and Lizards. East Lansing, MI, USA: Michigan State University Cooperative Extension Service; 1997. 94 p.

[13] Robert KA, Thompson MB. Influence of feeding on the metabolic rate of the lizard, *Eulamprus tympanum*. Copeia. 2000;(3):851–5.

[14] Secor SM, Boehm MC. Specific dynamic action of ambystomatid salamanders and the impact of meal size, meal type, and body temperature. Physiol Biochem Zool. 2006;79:720–35. 10.1086/505511 16826498

[15] Wang T, Burggren WW, Nobrega E. Metabolic, ventilatory, and acid-base responses associated with specific dynamic action in the toad *Bufo marinus*. Physiol Zool. 1995;68(2):192–205.

[16] McCue MD, Bennett AF, Hicks JW. The effect of meal composition on specific dynamic action in burmese pythons (*Python molurus*). Physiol Biochem Zoology. 10.1086/427049 15778938

[17] Crocker-Buta SP, Secor SM. Determinants and repeatability of the specific dynamic response of the corn snake, *Pantherophis guttatus*. Comp Biochem Physiology A. 2014;169:60–9. 10.1016/j.cbpa.2013.12.008 24361263

[18] Burggren WW, Infantino RL, Townsend DL. Developmental changes in cardiac and metabolic physiology of the direct-developing tropical frog *Eleutherodactylus coqui*. J Exp Biol. 1990; 152:129–47.

[19] Gore M, Burggren WW. Cardiac and metabolic physiology of early larval zebrafish (*Danio rerio*) reflects parental swimming stamina. Front Physiol. 2012;3:35 10.3389/fphys.2012.00035 22375123 PMC3285806

[20] Lighton JRB. Measuring Metabolic Rates: A Manual for Scientists. Oxford: Oxford University Press; 2008.

[21] Vleck D. Measurement of O_2_ consumption, CO_2_ production, and water vapor production in a closed system. J Appl Physiol. 1987;62(5):2103–6. 3110127 10.1152/jappl.1987.62.5.2103

[22] Hutchinson GE. Homage to Santa Rosalia or why are there so many kinds of animals. Am Nat. 1959;93:145–59.

[23] Murray IW, Fuller A, Lease HM, Mitchell D, Woldf BO, Hefem RS. Low field metabolic rates for geckos of the genus *Rhoptropus* may not be surprising. J Arid Environ. 2015;113:35–42.

[24] Iglesias S, Thompson MB, Seebacher F. Energetic cost of a meal in a frequent feeding lizard. Comp Biochem Phys A. 2003;135(3):377–82. 10.1016/s1095-6433(03)00076-x 12829046

[25] Angilletta MJ. Variation in metabolic rate between populations of a geographically widespread lizard. Physiol Biochem Zool. 2001;74(1):11–21. 10.1086/319312 11226010

[26] Zannoni JT. Measuring the Effects of Temperature and Nutritional State on Metabolic Rates of Eastern Fence Lizards, *Sceloporus undulate*. Akron, Ohio: University of Akron; 1997.

[27] Nagy KA, Huey RB, Bennett AF. Field energetics and foraging mode in Kalahari lactertid lizards. Ecology. 1984;65(2):588–96.

[28] Pan ZC, Ji X, Lu HL, Ma XM. Influence of food type on specific dynamic action of the Chinese skink *Eumeces chinensis*. Comp Biochem Physiol A. 2005;140(1):151–5. 10.1016/j.cbpb.2004.11.013 15664324

[29] Hare KM, Pledger S, Thompson MB, Miller JH, Daugherty CH. Daily patterns of metabolic rate among New Zealand lizards (Reptilia: Lacertilia: Diplodactylidae and Scincidae). Physiol Biochem Zool. 2006;79(4):745–53. 10.1086/504618 16826500

[30] Parker SL. Physiological ecology of the ground skink, *Scincella lateralis* in South Carolina: thermal biology, metabolism, water loss and seasonal patterns. Herp Conserv Biol. 2014;9(2):309–21.

[31] White CR, Kearney MR. Metabolic scaling in animals: methods, empirical results, and theoretical explanations. Comprehensive Physiol. 2014. 10.1002/cphy.c110049 24692144

[32] Glazier DS. Is metabolic rate a universal ‘pacemaker’for biological processes? Biol Reviews. 2015;90(2):377–407. 10.1111/brv.12115 24863680

[33] Agutter PS, Tuszynski JA. Analytic theories of allometric scaling. J Exp Biol. 2011; 214(7):1055–62. 21389188 10.1242/jeb.054502

[34] Zari TA. Seasonal metabolic acclimatization in the herbivorous desert lizard *Uromastyx philbyi* (Reptilia: Agamidea) from western Saudi Arabia. J Therm Biol. 2016;60:180–5. 10.1016/j.jtherbio.2016.07.014 27503731

[35] Bennett AF. The energetics of reptilian activity. Biology of the Reptilia. 1982;13:155–99.

[36] Andrews RM, Pough FH. Metabolism of squamate reptiles: allometric and ecological relationships. Physiol Zool. 1985:214–31.

[37] Al-Sadoon MK. Influence of a broad temperature range on the oxygen consumption rates of three desert lizard species. Comp Biochem Physiol A: 1986;84(2):339–44. 2873932 10.1016/0300-9629(86)90626-2

[38] Karasov WH, Anderson RA. Correlates of average daily metabolism of field-active zebra-tailed lizards (*Callisaurus draconoides*). Physiol Zool. 1998;71(1):93–105. 9472817 10.1086/515887

[39] Pelster B. Environmental influences on the development of the cardiac system in fish and amphibians. Comp Biochem Physiol A. 1999;124(4):407–12. 10.1016/s1095-6433(99)00132-4 10682238

[40] Burggren W, Roberts J. 9 Respiration and Metabolism. Comparative Animal Physiology, Environmental and Metabolic Animal Physiology. 1991:353.

[41] Clarke A, Johnston NM. Scaling of metabolic rate with body mass and temperature in teleost fish. J Anim Ecol. 1999;68(5):893–905.

[42] Hölker F. The metabolic rate of roach in relation to body size and temperature. J Fish Biol. 2003;62(3):565–79.

[43] Barrionuevo W, Burggren W. O_2_ consumption and heart rate in developing zebrafish (*Danio rerio*): influence of temperature and ambient O_2_. Am J Physiol. 1999;276(2):R505–R13. 9950931 10.1152/ajpregu.1999.276.2.R505

[44] Hochscheid S, Bentivegna F, Speakman JR. Long‐Term Cold Acclimation Leads to High Q_10_ Effects on Oxygen Consumption of Loggerhead Sea Turtles *Caretta caretta*. Physiol Biochem Zool. 2004;77(2):209–22. 10.1086/381472 15095241

[45] Carey C. Effect of constant and fluctuating temperatures on resting and active oxygen consumption of toads, *Bufo boreas*. Oecologia. 1979;39(2):201–12. 28309437 10.1007/BF00348069

[46] Al-Sadoon MK, Abdo NM. Temperature effects on oxygen consumption of two nocturnal geckos, *Ptyodactylus hasselquistii* (Donndorff) and *Bunopus tuberculatus* (Blanford) (Reptilia: Gekkonidae) in Saudi Arabia. J Comp Physiol B 1989;159:1–4.

[47] Watson CM L. G. The role of temperature in determining distributions and coexistence of three species of *Plestiodon*. J Therm Biol. 2012;37(5):374–9.

[48] Gause GF. The Struggle for Existence. Baltimore, MD, USA: William and Wilkins; 1934.

