# Supplementary material for: Interspecific Differences in Metabolic Rate and Metabolic Temperature Sensitivity Create Distinct Thermal Ecological Niches in Lizards (Plestiodon)
Source: PLoS One. 2016 Oct 19;11(10):e0164713. doi: 10.1371/journal.pone.0164713 (PMC5070829; doi:10.1371/journal.pone.0164713)
Supplement: S1 Table — (DOCX) [file pone.0164713.s001.docx]

|  | Mass (g) | *V*O2 (mL/g/h) | | |
| --- | --- | --- | --- | --- |
| *Plestiodon fasciatus* | | 20°C | 25°C | 30°C |
| A | 6.96 | 0.0551 | 0.1531 | 0.6072 |
| B | 6.97 | 0.0611 | 0.1805 | 0.5746 |
| C | 5.41 | 0.0801 | 0.1136 | 0.8694 |
| D | 5.15 | 0.0806 | 0.1312 | 0.6824 |
| E | 4.68 | 0.1114 | 0.1363 | 1.1958 |
| F | 4.25 | 0.1294 | 0.1573 | 1.2850 |
| *Plestiodon inexpectatus* | | 20°C | 25°C | 30°C |
| A | 8.61 | 0.0407 | 0.1421 | 0.2610 |
| B | 10.80 | 0.0442 | 0.2853 | 0.3182 |
| C | 18.29 | 0.0476 | 0.3011 | 0.5903 |
| D | 19.24 | 0.0495 | 0.3818 | 0.1581 |
| E | 7.18 | 0.0722 | 0.1791 | 0.4096 |
| F | 5.75 | 0.1276 | 1.2074 | 0.6257 |
| G | 5.83 | 0.5320 | 0.1462 | 0.3873 |
| *Plestidon laticeps* | | 20°C | 25°C | 30°C |
| A | 25.99 | 0.0382 | 0.0571 | 0.1498 |
| B | 17.65 | 0.0411 | 0.0954 | 0.1749 |
| C | 24.38 | 0.0414 | 0.0789 | 0.1748 |
| D | 22.90 | 0.0471 | 0.0893 | 0.1261 |
| E | 34.49 | 0.0505 | 0.0564 | 0.0902 |
| F | 26.68 | 0.0559 | 0.1047 | 0.1174 |
| G | 12.97 | 0.0629 | 0.1605 | 0.2881 |
